# *Helicobacter pylori*, herpes simplex virus‐1, varicella‐zoster virus, and dementia risk

**DOI:** 10.1002/dad2.70414

**Published:** 2026-07-06

**Authors:** Thomas J. Littlejohns, Wenyu Liu, Carol Brayne, Julia Butt, Jennifer A. Collister, Nita G. Forouhi, Effrossyni Gkrania‐Klotsas, Shabina Hayat, Katie Jeffery, Elżbieta Kuźma, Robert N. Luben, Alexander J. Mentzer, Julie Parsonnet, Nicholas Wareham, Tim Waterboer, David J. Hunter

**Affiliations:** ^1^ Nuffield Department of Population Health University of Oxford Oxford Oxfordshire UK; ^2^ Cambridge Public Health University of Cambridge Cambridge Cambridgeshire UK; ^3^ German Cancer Research Center (DKFZ) Heidelberg Germany; ^4^ MRC Epidemiology Unit, Institute of Metabolic Science University of Cambridge School of Clinical Medicine Cambridge Cambridgeshire UK; ^5^ Department of Medicine University of Cambridge Cambridge Cambridgeshire UK; ^6^ Department of Behavioural Science and Health University College London, Greater London London UK; ^7^ Oxford University Hospitals NHS Foundation Trust Oxford Oxfordshire UK; ^8^ Radcliffe Department of Medicine University of Oxford Oxford Oxfordshire UK; ^9^ Albertinen Krankenhaus/Albertinen Haus gGmbH, Academic Teaching Hospital of the Faculty of Medicine University of Hamburg Hamburg Germany; ^10^ NIHR Biomedical Research Centre Moorfields Eye Hospital NHS Foundation Trust and UCL Institute of Ophthalmology, Greater London London UK; ^11^ The Centre for Human Genetics, Nuffield Department of Medicine University of Oxford Oxford Oxfordshire UK; ^12^ Chinese Academy of Medical Sciences Oxford Institute University of Oxford Oxford Oxfordshire UK; ^13^ Division of Infectious Diseases and Geographic Medicine, Department of Medicine Stanford University Stanford California USA; ^14^ Department of Epidemiology and Population Health Stanford University Stanford California USA; ^15^ Department of Epidemiology Harvard TH Chan School of Public Health Boston Massachusetts USA

**Keywords:** dementia, European Prospective Investigation into Cancer in Norfolk (EPIC‐Norfolk), *Helicobacter pylori*, herpes simplex‐1, longitudinal, varicella‐zoster virus

## Abstract

**Introduction:**

*Helicobacter pylori* (*H. pylori*), herpes simplex‐1 (HSV‐1), and varicella‐zoster virus (VZV) could increase dementia risk; however, evidence from large cohorts with long‐term follow‐up is scarce.

**Methods:**

Multivariable‐adjusted Cox proportional‐hazards models were used to investigate the association between *H. pylori*, HSV‐1, and VZV seropositivity and dementia risk over 24 years in 8550 participants from the European Prospective Investigation into Cancer in Norfolk study in the United Kingdom.

**Results:**

*H. pylori* (hazard ratio [HR] = 1.24, 95% confidence interval [CI]: 1.09 to 1.41), but not HSV‐1 (HR = 1.03, 95% CI: 0.91 to 1.18) or VZV (HR = 1.01, 95% CI: 0.86 to 1.19), was associated with dementia risk. The *H. pylori*–dementia association remained robust among incident cases >15 years after blood draw (HR = 1.26, 95% CI: 1.06 to 1.48). Seropositive tertiles of the *H. pylori* antibodies CagA (*p *= 0.007) and GroEL (*p *= 0.001) demonstrated significant trends with dementia risk.

**Discussion:**

*H. pylori* may represent a novel dementia prevention target, although early‐life socioeconomic factors might confound the association.

## HIGHLIGHTS

- Exposure to certain pathogens might be related to dementia risk.
- *H. Pylori*, but not HSV‐1 or VZV, was associated with an increased risk of dementia.
- The association remained similar after excluding cases diagnosed within 15 years.
- *H. pylori* antibodies CagA and GroEL were positively associated with dementia risk.

## 1 BACKGROUND

There is evidence that some infectious pathogens could increase dementia risk, in particular certain members of the Herpesviridae family, herpes simplex virus‐1 (HSV‐1) and varicella‐zoster virus (VZV), and the bacterium *Helicobacter pylori* (*H. pylori*).^1^, ^2^ These infections occur throughout the life course and are common, with a seroprevalence in Western populations of approximately 60%,^3^ 90%,^4^ and 40%^5^ for HSV‐1, VZV, and *H. pylori*, respectively. HSV‐1 and VZV are neurotrophic viruses, and reactivation can cause peripheral and central nervous system complications, including myelitis and vasculopathy.^6^ *H. pylori* can cause chronic atrophic gastritis and could adversely affect brain health via increased inflammatory responses and malabsorption of essential nutrients.^2^

Meta‐analyses have found that clinically diagnosed HSV‐1 and VZV are associated with dementia risk; however, these are largely based on retrospective and case‐control studies without serological ascertainment of the exposures.^7^, ^8^ In contrast, prospective studies on HSV‐1 and VZV seropositivity and dementia risk have produced inconsistent findings, with some finding positive associations^9^, ^10^, ^11^, ^12^ and others finding null associations.^13^, ^14^, ^15^, ^16^ However, generally these studies have consisted of a few hundred incident dementia cases with short‐follow‐up between infectious disease serology measurement and a diagnosis of dementia. A more consistent association between *H. pylori* seropositivity and an increased risk of dementia has been observed in population‐based longitudinal studies.^17^, ^18^, ^19^, ^20^ Certain strains of *H. pylori* are more virulent, with CagA strongly related to risk of gastric cancer and vascular complications.^21^, ^22^ However, previous studies did not investigate whether specific *H. pylori* antibody levels were differentially associated with dementia risk.

We address these gaps by investigating the association between *H. pylori*, HSV‐1, and VZV seropositivity and antibody levels with dementia risk in a large cohort with more than a thousand dementia cases occurring over 24 years of follow‐up. We also examined whether age as well as apolipoprotein E (APOE) ε4, the strongest genetic risk factor for dementia, modified the association between *H. pylori*, HSV‐1, and VZV and dementia risk.

## 2 METHODS

### 2.1 EPIC‐Norfolk

The European Prospective Investigation into Cancer in Norfolk (EPIC‐Norfolk) study is a population‐based cohort of 25,639 women and men aged 40 to 79 years recruited between 1993 and 1997.^23^ Participants completed a postal health and lifestyle questionnaire and underwent an assessment, termed Health Check 1 (HC1), which included physical examinations and blood sample collection. Of these, 15,028 participants, as well as 758 participants who did not attend HC1, attended Health Check 2 (HC2) between 1998 and 2000 (*n* = 15,786). The data collection from HC1 was repeated at HC2 and additional measures were obtained, including infectious agents measured using the HC2 blood samples.^24^ Informed consent was obtained from all participants. The EPIC‐Norfolk study was approved by the Norfolk Local Research Ethics Committee (05/Q0101/191) and East Norfolk and Waveney National Health Service (NHS) Research Governance Committee (2005EC07L). EPIC‐Norfolk also has approval for follow‐up through record linkage (REC ref 98CN01).

### 2.2 Infectious diseases

Antibody responses to antigens of several infectious agents were measured using a quantitative multiplex serology panel on serum samples from approximately 10,000 randomly selected participants who attended HC2. Assay development, implementation, and quality control were performed at the German Cancer Research Center (DKFZ) in Heidelberg. Briefly, antigens of infectious agents were recombinantly expressed as glutathione‐S‐transferse (GST)‐tagged proteins and affinity‐purified on fluorescently labeled beads (Luminex, Diasorin, Italy). A suspension array of these antigen‐loaded beads was then incubated with serum, and antigen‐bound serum antibodies were labeled by a biotinylated secondary anti‐human IgM/IgA/IgG antibody and a reporter fluorescence (Streptavdin‐R‐phycoerythrin). A Luminex analyzer quantified the amount of bound serum antibody as median fluorescence intensity (MFI) of at least 100 beads per type and, consequently, antigen.^25^ Participants were classified as seropositive if antibody responses to ≥2 out of five *H. pylori* antigens, ≥2 out of two HSV‐1 antigens, and ≥1 out of two VZV antigens reached defined seroreactive MFI thresholds (Table S1). The prespecified algorithms to determine seropositivity were developed by the DKFZ team based on various inspections of the data and previously validated work.^26^, ^27^ The DKFZ team was not aware of the dementia status of participants. The panel also included antigens for HSV‐2, Epstein‐Barr virus (EBV), human cytomegalovirus (HCMV), human herpes virus 6a (HHV6a), human herpes virus 6b (HHV6b), human herpes virus 6 (HHV6), human herpes virus 7 (HHV7), and *Toxoplasma gondii* (*T. gondii*).

Between 2002 and 2006, participants responded to a follow‐up questionnaire that included the question *Are there any other infections that you commonly get more than once or twice a year?*, with multiple‐choice options including “cold sores” and “shingles.” This was used to derive variables for evidence of reactivation by classifying participants into categorical variables each consisting of three groups for HSV‐1 (1. seronegative; 2. seropositive without cold sores; 3. seropositive with cold sores) and VZV (1. seronegative; 2. seropositive without shingles; 3. seropositive with shingles). Seronegative participants who self‐reported having cold sores (*n* = 20) or shingles (*n* = 8) were excluded from the HSV‐1 and VZV analyses, respectively.

### 2.3 Dementia

Dementia was ascertained through the following electronic medical records, which were available cohort‐wide: hospital inpatient records obtained from Hospital Episode Statistics, death registry records; and the Mental Health Minimum Data Set, the Mental Health and Learning Disabilities Data Set, and the Mental Health Services Data Set.^28^ The latter three mental health datasets included information on individuals in contact with mental health services and memory clinics. All diagnoses were recorded using the International Classification of Diseases coding system (see Table S2 for diagnostic codes).^28^

**RESEARCH IN CONTEXT**

1. **Systematic review**: A structured search in MEDLINE was used to identify longitudinal studies on the relationship between H. pylori, HSV‐1, and VZV and dementia risk. Meta‐analyses have produced mixed findings, and there is a lack of studies that have used serological exposure measures in large cohorts with long follow‐up periods.
2. **Interpretation**: H. pylori seropositivity was associated with an increased risk of dementia in 8550 participants (n = 1127 cases) over a median of 21.6 years of follow‐up. The associations appear to be driven by the H. pylori antibodies CagA and GroEL, which demonstrated linear positive associations with dementia risk. No association between HSV‐1 and VZV and dementia risk was observed.
3. **Future directions**: Early‐life socioeconomic factors could confound the association between H. pylori and dementia risk, and this warrants further exploration in future studies.

### 2.4 Covariates

We selected sociodemographic, lifestyle, and health‐related covariates, which could potentially confound the associations between infectious pathogens and dementia risk.^24^, ^29^, ^30^ A health and lifestyle questionnaire was used to obtain the following information at HC2: age in years, sex (male, female), education (degree or equivalent, upper secondary school (A‐level) or equivalent, lower secondary school (O‐level) or equivalent, less than lower secondary school (O‐level), or no qualifications), occupational social class (l – professionals; ll – managerial and technical; lllN + IIIM – non‐manual and manual skilled; IV – partly skilled; V – unskilled), smoking status (never, former, current), and alcohol drinking status (never, former, current). Physical activity was derived using a four‐level activity index from a validated questionnaire designed to assess combined work and leisure activity.^31^ Levels of activity were defined as active (sedentary job with > 1 h recreational activity per day, standing job with >0.5 h recreational activity per day, physical job with at least some recreational activity, or a heavy manual job); moderately active (sedentary job with 0.5 to 1.0 h recreational activity per day, standing job with <0.5 h recreational activity per day, or physical job with no recreational activity); moderately inactive (sedentary job with <0.5 h recreational activity per day or standing job with no recreational activity); or inactive (sedentary job and no recreational activity). The prevalence of hypertension (no, yes) was derived from a combination of self‐reported doctor diagnosis, self‐reported use of antihypertensive medication, or mean of two blood pressure measurements^32^ of >140 or >90 for systolic and diastolic blood pressure, respectively. The prevalence of diabetes (no, yes) was derived from a combination of self‐reported doctor diagnosis or self‐reported use of diabetic medication. The Townsend deprivation score (in quintiles) was used as an indicator of material deprivation and was assigned to each participant corresponding to the output area of their residential postcode using small area data from the 1991 Census.^33^ Body mass index (BMI, kg/m^2^) was derived from weight (kg) using scales and standing height (m) measured during the physical examinations (<25; 25 to 29.9; ≥30). APOE ε4 carrier status (ε 4 non‐carrier; ε4 carrier) was derived using rs429358 and rs7412 single‐nucleotide polymorphisms, which were directly genotyped using DNA extracted from blood collected in EDTA tubes at the second health check. For participants who did not provide blood samples at HC2, DNA for genotyping was extracted from remnant buffy coats collected at HC1. APOE genotype was assessed using pyrosequencing.^34^

### 2.5 Statistical analysis

Descriptive statistics were used to compare baseline characteristics between participants by *H. pylori*, HSV‐1, and VZV serostatus. Multivariable Cox proportional‐hazards regression models were used to assess the association between *H. pylori*, HSV‐1, and VZV seropositivity, compared to seronegativity, with risk of dementia. Follow‐up time in years was calculated from date of attending HC2 to whichever censoring date occurred first: (1) date of dementia diagnosis, (2) date of death, or (3) end of follow‐up date. End of follow‐up was defined as March 31, 2022, as this was the last date of medical record availability for the whole cohort. The proportional‐hazards assumption was tested by visually assessing the scaled Schoenfeld residuals. Models were fully adjusted for age, sex, education and occupational social class, Townsend deprivation score, BMI, smoking status, alcohol intake, physical activity, hypertension, and diabetes. To investigate the impact of adjustment, models were repeated with incremental inclusion of each covariate. Participants with missing data for the covariates were treated as a separate stratum of the covariate. Multicollinearity among the covariates was investigated using a variance inflation factor.

To account for reverse causation due to the fact that dementia has a long pre‐clinical period,^35^ the association between *H. pylori*, HSV‐1, and VZV with dementia risk was repeated by restricting the follow‐up period to ≤15 and > 15 years of follow‐up. To investigate the role of effect modification, we tested for interactions between *H. pylori*, HSV‐1, and VZV by (1) age (50 to 64, ≥65 years), (2) birth year (< 1930, 1930 to 1939, ≥1940), (3) sex (male, female), and (4) APOE ε4 carrier status (no, yes) in association with dementia risk and subsequently repeated the main analyses within each stratum of age, birth year, sex, and APOE ε4 carrier status.

In secondary analyses, we investigated whether natural log‐transformed individual antibody levels for *H. pylori*, HSV‐1, and VZV displayed a dose–response association with dementia risk. Seronegative status (the reference group) was defined as being below the MFI cut point for the antibody of interest, while those above the cut points were categorized into seropositive tertiles (Table S1).

We investigated whether reactivation of HSV‐1, based on self‐reported cold sores, and VZV, based on self‐report shingles, was associated with dementia risk. These data were collected at a follow‐up questionnaire conducted between 2002 and 2006. For these analyses, baseline was defined as the date of follow‐up questionnaire completion and was restricted to participants without prevalent dementia at this time‐point. Finally, we investigated whether seropositivity and individual antibodies for the remaining infectious diseases on the serology panel were associated with dementia risk, including HSV‐2, EBV, HCMV, HHV6a, HHV6b, HHV6, HHV7, and *T. gondii*.

*P* values were two‐sided, and the type I error rate for statistical significance was set at *α* = .05.

Analyses were performed using Stata SE version 17.0 (StataCorp) and R version 4.4.0.

## 3 RESULTS

A total of 9205 participants had infectious disease serology data available. We excluded participants with prevalent dementia (*N* = 1), those aged less than 50 to restrict the sample to participants at risk of dementia over the follow‐up period (*N* = 600), and those without an end of follow‐up date based on the available medical record data (*N* = 61). The final sample size was 8550 participants (Figure S1).

Of these, 2288 (26.8%), 5480 (64.1%), and 7055 (82.5%) participants were seropositive for *H. pylori*, HSV‐1, and VZV, respectively. Seropositive participants for each infectious disease were, on average, older, had lower education, and were more likely to be hypertensive (Table 1). Participants seropositive for *H. pylori* or VZV, but not HSV‐1, were more likely to be men. Participants seropositive for *H. pylori* or HSV‐1, but not VZV, were more likely to be from a lower social occupational class, have higher BMI, and be a former or current smoker.

**TABLE 1:** Baseline characteristics by *H. pylori*, HSV‐1, and VZV serostatus in EPIC‐Norfolk.

	Total sample	*H. pylori* serostatus, *N* (%)		HSV‐1 serostatus, *N* (%)		VZV serostatus, *N* (%)	
Characteristics	*N* (%) (*N* = 8550)	Seronegative (*N* = 6262)	Seropositive (*N* = 2288)	Seronegative (*N* = 3070)	Seropositive (*N* = 5480)	Seronegative (*N* = 1495)	Seropositive (*N* = 7055)
Age, mean (SD)	63.2 (8.3)	62.2 (8.2)	65.9 (8.1)	62.1 (8.2)	63.8 (8.3)	62.7 (8.4)	63.3 (8.3)
Sex							
Women	4921 (58)	3754 (60)	1167 (51)	1726 (56)	3195 (58)	987 (66)	3934 (56)
Men	3629 (42)	2508 (40)	1121 (49)	1344 (44)	2285 (42)	508 (34)	3121 (44)
Educational attainment							
Degree or equivalent	1162 (14)	933 (15)	229 (10)	531 (17)	631 (12)	210 (14)	952 (14)
Upper secondary school (A‐Level) or equivalent	3529 (41)	2611 (42)	918 (40)	1335 (44)	2194 (40)	647 (43)	2882 (41)
Lower secondary school (O‐Level) or equivalent	917 (11)	705 (11)	212 (9)	338 (11)	579 (11)	157 (11)	760 (11)
Less than lower secondary school (O‐level) or no qualifications	2939 (34)	2010 (32)	929 (41)	865 (28)	2074 (38)	480 (32)	2459 (35)
Missing	3 (<0.1)	3 (<0.1)	0 (0)	1 (<0.1)	2 (<0.1)	1 (<0.1)	2 (<0.1)
Townsend deprivation score, quintile							
1 (least deprived)	1705 (20)	1243 (20)	462 (20)	584 (19)	1121 (21)	295 (20)	1410 (20)
2	1705 (20)	1252 (20)	453 (20)	627 (20)	1078 (20)	325 (22)	1380 (20)
3	1705 (20)	1271 (20)	434 (19)	652 (21)	1053 (19)	295 (20)	1410 (20)
4	1704 (20)	1262 (20)	442 (19)	640 (21)	1064 (19)	280 (19)	1424 (20)
5 (most deprived)	1704 (20)	1215 (19)	489 (21)	556 (18)	1148 (21)	296 (20)	1408 (20)
Missing	27 (<1)	19 (<1)	8 (<1)	11 (<1)	16 (<1)	4 (<1)	23 (<1)
Occupational social class							
l – Professional	614 (7)	462 (7)	152 (7)	287 (9)	327 (6)	119 (8)	495 (7)
ll – Managerial and technical	3259 (38)	2453 (39)	806 (35)	1210 (39)	2049 (37)	583 (39)	2676 (38)
lllN + lllM – Non‐manual and manual skilled	3202 (37)	2316 (37)	886 (39)	1124 (37)	2078 (38)	542 (36)	2660 (38)
IV – Partly skilled	1046 (12)	743 (12)	303 (13)	314 (10)	732 (13)	181 (12)	865 (12)
V – Unskilled	271 (3)	183 (3)	88 (4)	74 (2)	197 (4)	47 (3)	224 (3)
Missing	158 (2)	105 (2)	53 (2)	61 (2)	97 (2)	23 (2)	135 (2)
BMI							
<25	3027 (35)	2262 (36)	765 (33)	1162 (38)	1865 (34)	570 (38)	2457 (35)
25 to 29.9	4067 (48)	2963 (47)	1104 (48)	1448 (47)	2619 (48)	655 (44)	3412 (48)
≥30	1435 (17)	1022 (16)	413 (18)	451 (15)	984 (18)	267 (18)	1168 (17)
Missing	21 (< 1)	15 (< 1)	6 (< 1)	9 (< 1)	12 (< 1)	3 (< 1)	18 (< 1)
Smoking status							
Never	4176 (49)	3211 (51)	965 (42)	1647 (54)	2529 (46)	760 (51)	3416 (48)
Former	3692 (43)	2566 (41)	1126 (49)	1209 (39)	2483 (45)	610 (41)	3082 (44)
Current	616 (7)	437 (7)	179 (8)	193 (6)	423 (8)	112 (8)	504 (7)
Missing	66 (<1)	48 (<1)	18 (<1)	21 (<1)	45 (<1)	13 (<1)	53 (<1)
Alcohol drinking status							
Never	394 (5)	262 (4)	132 (6)	132 (4)	262 (5)	68 (5)	326 (5)
Former	1489 (17)	1099 (18)	390 (17)	525 (17)	964 (18)	273 (18)	1216 (17)
Current	6473 (76)	4772 (76)	1701 (74)	2353 (78)	4120 (75)	1116 (75)	5357 (76)
Missing	194 (2)	129 (2)	65 (3)	60 (2)	134 (2)	38 (3)	156 (2)
Physical activity^*^							
Active	2715 (32)	1959 (31)	756 (33)	956 (31)	1759 (32)	464 (31)	2251 (32)
Moderately active	1957 (23)	1473 (24)	484 (21)	725 (24)	1232 (23)	311 (21)	1646 (23)
Moderately inactive	3071 (36)	2287 (37)	784 (34)	1113 (36)	1958 (36)	580 (39)	2491 (35)
Inactive	684 (8)	457 (7)	227 (10)	239 (8)	445 (8)	121 (8)	563 (8)
Missing	123 (1)	86 (1)	37 (2)	37 (1)	86 (2)	19 (1)	104 (2)
Hypertension							
No	3764 (44)	2845 (45)	919 (40)	1423 (46)	2341 (43)	703 (47)	3061 (43)
Yes	4786 (56)	3417 (55)	1369 (60)	1647 (54)	3139 (57)	792 (53)	3994 (57)
Diabetes							
No	7402 (87)	5463 (87)	1939 (85)	2708 (88)	4694 (86)	1293 (87)	6109 (87)
Yes	270 (3)	180 (3)	90 (4)	88 (3)	182 (3)	38 (3)	232 (3)
Missing	878 (10)	619 (10)	259 (11)	274 (9)	604 (11)	164 (69)	714 (10)
APOE ε4 carrier							
No	5955 (70)	4347 (69)	1608 (70)	2148 (70)	3807 (70)	1025 (69)	4930 (70)
Yes	2370 (28)	1758 (28)	612 (27)	841 (28)	1529 (28)	440 (29)	1930 (27)
Missing	225 (3)	157 (3)	68 (3)	81 (3)	144 (3)	30 (2)	195 (3)
Year of Birth							
1910 to 1920	118 (1)	75 (1)	43 (2)	38 (1)	80 (2)	25 (2)	93 (1)
1920 to 1930	2289 (27)	1418 (23)	871 (38)	692 (23)	1597 (29)	370 (25)	1919 (27)
1930 to 1940	3094 (36)	2237 (36)	857 (38)	1089 (36)	2005 (37)	518 (35)	2576 (37)
1940 to 1950	3010 (35)	2498 (40)	512 (22)	1232 (4)	1778 (32)	570 (38)	2440 (35)
1950 to 1960	39 (1)	34 (1)	5 (< 1)	19 (1)	20 (< 1)	12 (1)	27 (< 1)

*Note*: Overall, 82% of the study population had complete data.

Abbreviations: BMI, body mass index; *H. pylori*, *Helicobacter pylori*; HSV‐1, herpes simplex virus‐1; *N*, number of participants; SD, standard deviation; VZV, varicella‐zoster virus (%) refers to column percentages.

* Levels of activity were defined as follows: active (sedentary job with >1 h recreational activity per day, standing job with >0.5 h recreational activity per day, physical job with at least some recreational activity, or heavy manual job); moderately active (sedentary job with 0.5 to 1.0 h recreational activity per day, standing job with <0.5 h recreational activity per day, or physical job with no recreational activity); moderately inactive (sedentary job with <0.5 h recreational activity per day or standing job with no recreational activity); or inactive (sedentary job and no recreational activity).

**FIGURE 1:**
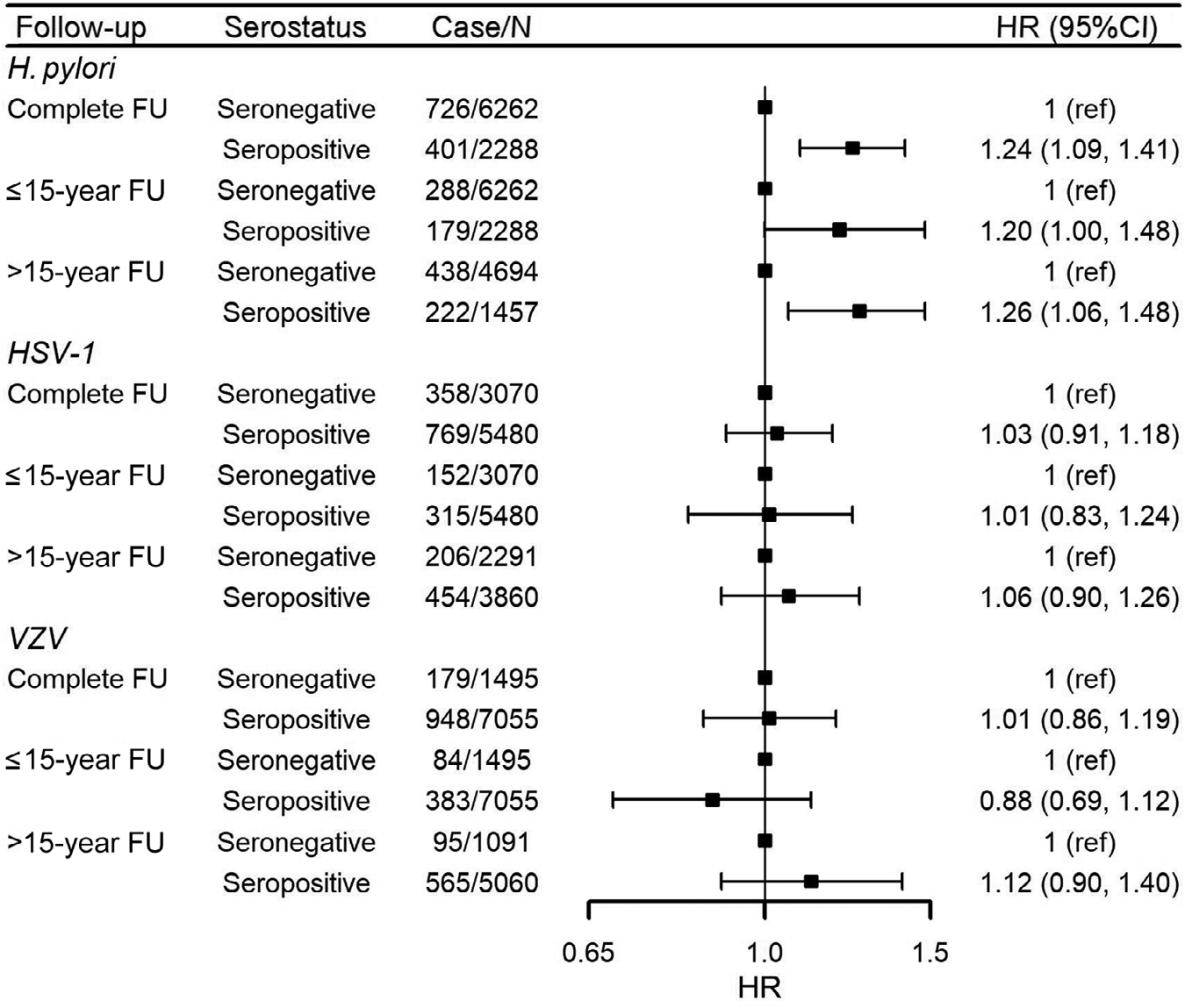
Association between *H. pylori*, HSV‐1, and VZV serostatus and risk of incident dementia by follow‐up length in EPIC‐Norfolk. CI, confidence interval; FU, follow‐up; HR, hazard ratio; HSV‐1, herpes simplex virus‐1; *H. pylori*, *Helicobacter pylori*; *N*, number of participants; ref, reference group; VZV, varicella‐zoster virus. Models adjusted for age, sex, education, occupational social class, Townsend deprivation score, body mass index, smoking status, alcohol drinking status, physical activity, hypertension, and diabetes.

Over a median of 21.6 years of follow‐up, 1127 participants developed dementia. The median age of dementia diagnosis was 85.6 years old (interquartile range = 81.0 to 89.7 years old; Figure S2), and the median length of follow‐up to diagnosis was 16.3 years (interquartile range = 12.3 to 19.6 years; Figure S3). In fully adjusted models, seropositivity for *H. pylori* (hazard ratio [HR] = 1.24, 95% confidence interval [CI]: 1.09 to 1.41), but not HSV‐1 (HR = 1.03, 95% CI: 0.91 to 1.18) or VZV (HR = 1.01, 95% CI: 0.0.86 to 1.19), was associated with an increased risk of dementia (Figure 1). Incremental adjustment for covariates demonstrated that, as expected, age had a substantial impact on the effect estimates, while the HRs remained similar when more covariates were incorporated (Table S3). In fully adjusted models, after restricting the analysis to dementia cases occurring 15 years after baseline (*n* = 660 cases), the association between *H. pylori* and incident dementia remained consistent (HR = 1.26, 95% CI: 1.06 to 1.48), while the risk of dementia remained null for HSV‐1 (HR = 1.06, 95% CI: 0.90 to 1.26) and VZV (HR = 1.12, 95% CI: 0.90 to 1.40; Figure 1).

The HRs and 95% CIs for the associations between the covariates included in the fully adjusted model and dementia risk are provided in Table S4. Of these, age in years (HR = 1.17, 95% CI: 1.16 to 1.18), upper secondary school level of educational attainment compared to degree or equivalent level (HR = 1.30, 95% CI: 1.04 to 1.64), being a former smoker compared to never smokers (HR = 1.15, 95% CI: 1.00 to 1.31), physical inactivity compared to high physical activity (HR = 1.24, 95% CI: 1.00 to 1.53), hypertension (HR = 1.14, 95% CI: 1.00 to 1.31), diabetes (HR = 1.86, 95% CI: 1.40 to 2.48), and APOE ε4 carrier (HR = 2.45, 95% CI: 2.17 to 2.76) were associated with dementia risk.

**FIGURE 2:**
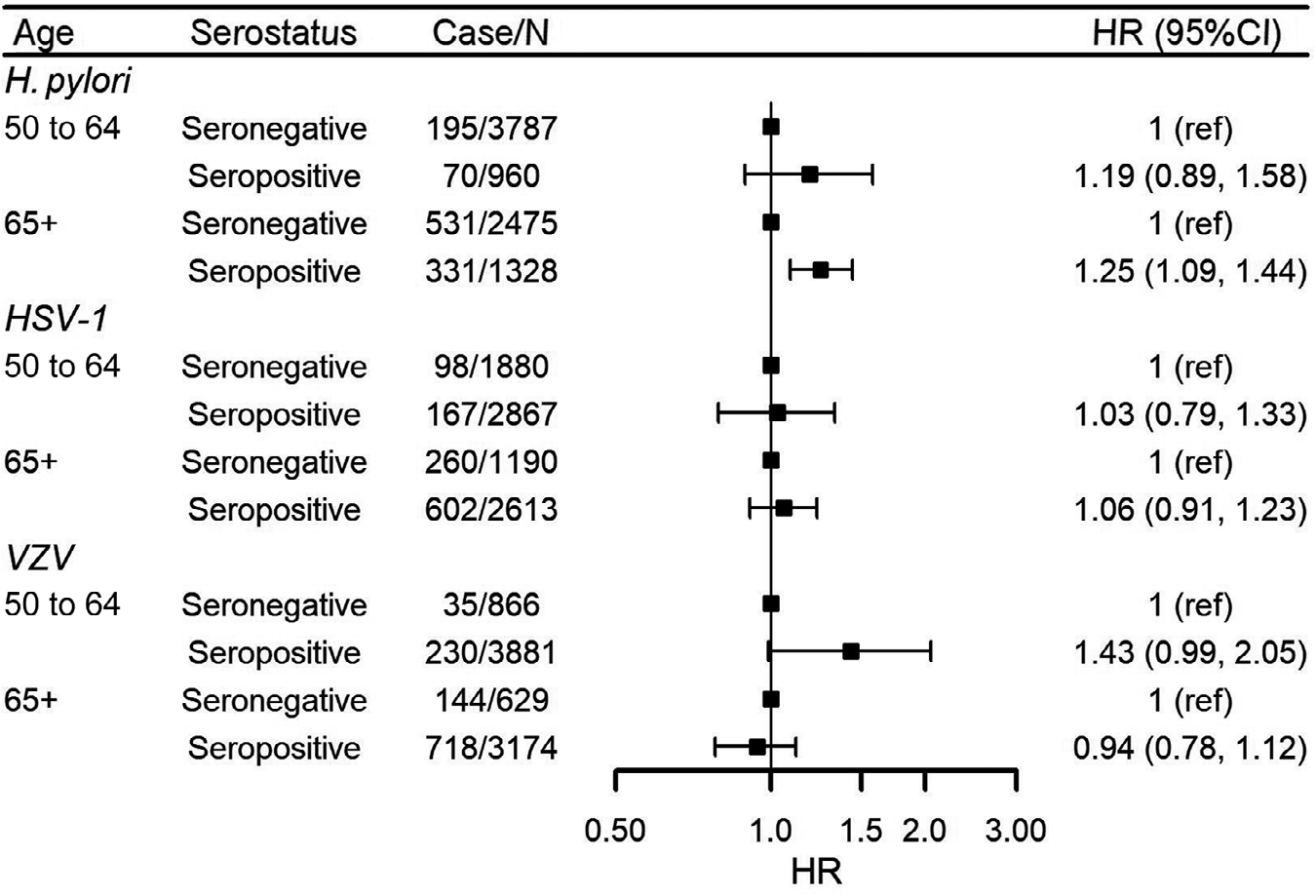
Association between *H. pylori*, HSV‐1, and VZV serostatus and risk of incident dementia by baseline age in EPIC‐Norfolk. CI, confidence interval; HR, hazard ratio; HSV‐1, herpes simplex virus‐1; *H. pylori*, *Helicobacter pylori*; *N*, number of participants; ref, reference group; VZV, varicella‐zoster virus. Models adjusted for age, sex, education, occupational social class, Townsend deprivation score, body mass index, smoking status, alcohol drinking status, physical activity, hypertension, and diabetes. *p* value for interaction between age groups and *H. pylori* = 0.61, age groups and HSV‐1 = 0.89, and age groups and VZV = 0.04.

In models stratified by baseline age, an increased risk of dementia was observed in participants who were *H. pylori* seropositive at ≥65 years old (HR = 1.25, 95% CI: 1.09 to 1.44), but not in those 50 to 64 years old (HR = 1.19, 95% CI: 0.89 to 1.58; Figure 2). HSV‐1 and VZV were not significantly associated with dementia in any age group, although the association between VZV seropositivity at 50 to 64 years old and dementia was borderline significant (HR = 1.43, 95% CI: 0.99 to 2.05), and a statistically significant interaction between VZV and age was observed (*p* value for interaction = 0.04). No statistically significant interactions between age and *H. pylori* (*p* value for interaction = 0.61) and age and HSV‐1 (*p* value for interaction = 0.93) were found. Similar associations to age were observed when stratifying by birth year (Table S5). The associations between *H. pylori* and dementia were slightly stronger in males (HR = 1.35, 95% CI: 1.11 to 1.64), though the direction remained the same in females (HR = 1.19, 95% CI: 1.00 to 1.40), and the *p* value for interaction between sex and *H. pylori* was non‐significant (*p = *0.35, Table S6). No associations between HSV‐1 and VZV with dementia risk were observed by sex.

**FIGURE 3:**
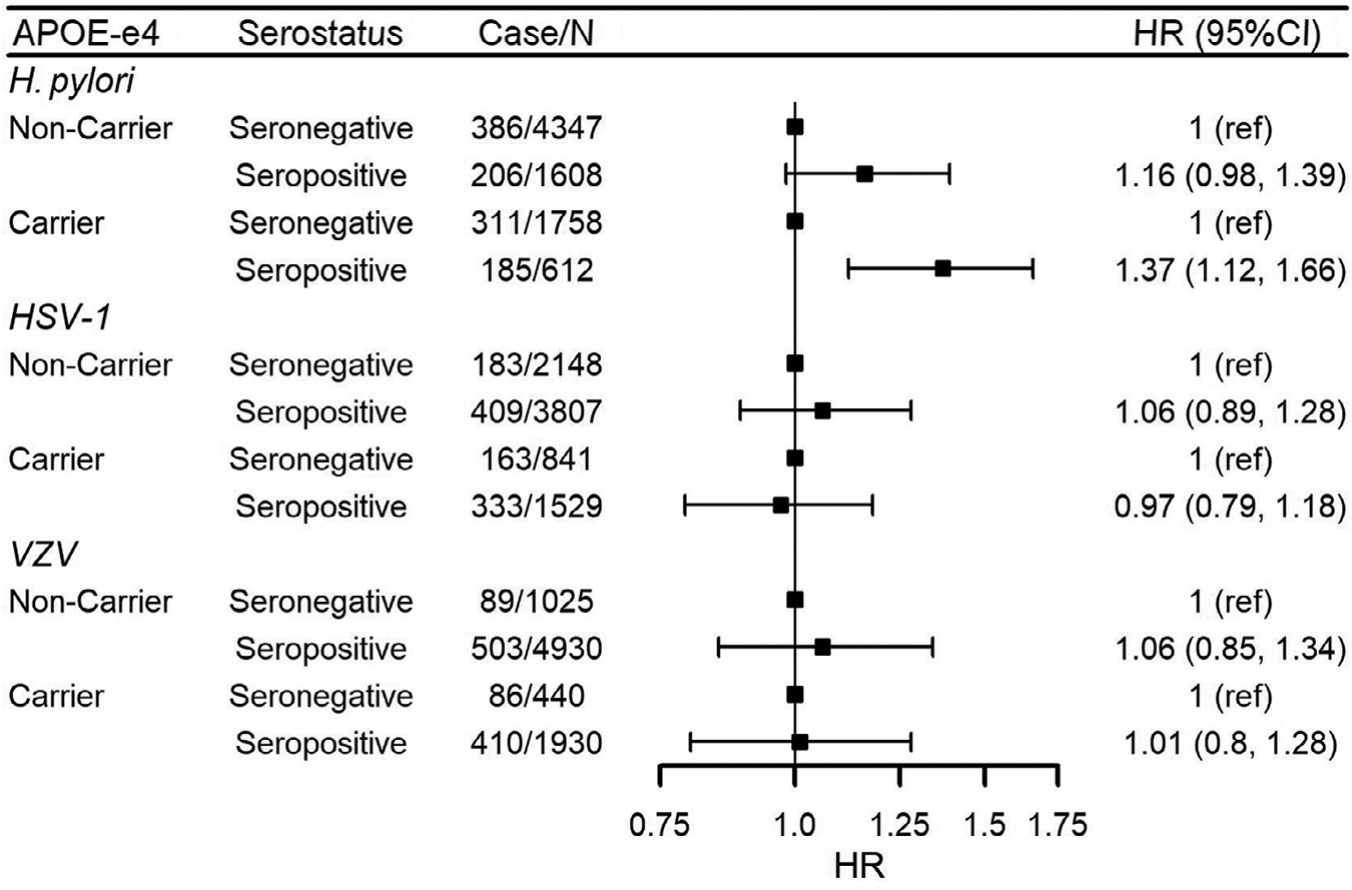
Association between *H. pylori*, HSV‐1, and VZV serostatus and risk of incident dementia by APOE ε4 status in EPIC‐Norfolk. CI, confidence interval; HR, hazard ratio; HSV‐1, herpes simplex virus‐1; *H. pylori*, *Helicobacter pylori*; *N*, number of participants; ref, reference group; VZV, varicella‐zoster virus. Models adjusted for age, sex, education, occupational social class, Townsend deprivation score, body mass index, smoking status, alcohol drinking status, physical activity, hypertension, and diabetes. *p* value for interaction between APOE ε4 status and HSV‐1 = 0.33, APOE ε4 status and VZV = 0.47, and APOE ε4 status and *H. pylori* = 0.15.

In models stratified by APOE ε4carrier status, the direction of associations between *H. pylori* and dementia risk remained similar among APOE ε4 carriers (HR = 1.37, 95% CI: 1.12 to 1.66) and non‐APOE ε4 carriers (HR = 1.16, 95% CI: 0.98 to 1.39, Figure 3). However, the interaction between APOE and *H. pylori* was non‐significant (*p *= 0.29). Similar to the main analyses, HSV‐1 (*p* value for interaction = 0.23) and VZV (*p* value for interaction  = 0.57) were not associated with dementia risk regardless of APOE ε4 carrier status.

**FIGURE 4:**
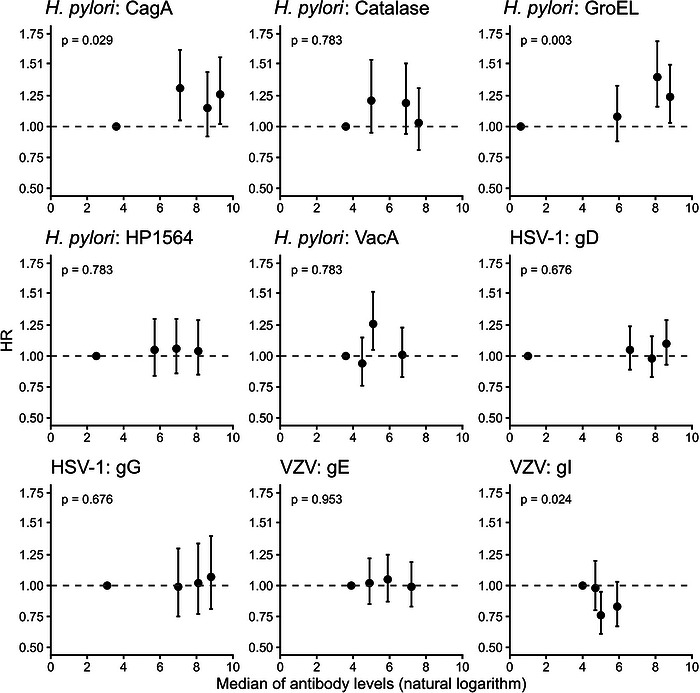
Association between *H. pylori*, HSV‐1, and VZV antibodies in tertiles among seropositive participants and risk of incident dementia compared to seronegative participants in EPIC‐Norfolk. HR, hazard ratio; HSV‐1, herpes simplex virus‐1; *H. pylori*, *Helicobacter pylori*; *N*, number of participants; ref, reference group; VZV, varicella‐zoster virus. Models adjusted for age, sex, education, occupational social class, Townsend deprivation score, body mass index, smoking status, alcohol drinking status, physical activity, hypertension, and diabetes. *P* value for test of linear trend is 0.42 for gD, 0.34 for gG, 0.95 for gE, 0.01 for gI, 0.007 for CagA, 0.26 for catalase, 0.001 for GroEL, 0.54 for HP1564, and 0.27 for VacA.

Antibody level distributions are shown in Figure S4. Using tests for linear trends, *H. pylori*‐CagA (*p* = 0.007) and *H. pylo*ri‐GroEL (*p* = 0.001) were associated with an increased dementia risk, while VZV‐gl (*p* = 0.01) was inversely associated with dementia (Figure 4). No statistically significant tests for linear trends were observed between antibody levels for HSV1‐gD (p = 0.42), HSV1‐gG (*p* = 0.34), VZV‐gE (*p* = 0.95), *H. pylori*‐catalase (*p* = 0.26), *H. pylori*‐HP1564 (*p* = 0.54), or *H‐pylori*‐VacA (*p* = 0.27) and dementia.

Reactivated HSV‐1 or VZV, based on self‐reported cold sores or shingles, was not associated with dementia risk shingles, respectively (Figure S5). No associations between HSV‐2, EBV, HCMV, HHV6a, HHV6b, HHV6, and *T. gondii* seropositivity and dementia risk were observed, while HHV7 was inversely associated with dementia risk (HR = 0.86, 95% CI: 0.74 to 0.99; Table S7). Of the individual antibodies, only gene product p52 (UL44) response for HCMV was associated with dementia risk, with a significant positive association observed (*p* = 0.02).

## 4 DISCUSSION

We found that *H. pylori* seropositivity was associated with a 24% increased risk of developing dementia over a 24‐year follow‐up period. This association remained robust when the analysis was restricted to dementia cases diagnosed more than 15 years after *H. pylori* measurement and was strongest among those participants aged ≥65 years old at baseline and among those with a genetic predisposition to dementia based on APOE ε4 carrier status. Of the five *H. pylori* antigens measured to determine serostatus, CagA and GroEL were individually associated with an increased risk of dementia, whereas catalase, HP1564, and VacA were not associated with dementia risk. In contrast, we found no association between HSV‐1 and VZV seropositivity or their individual antibodies and risk of dementia.

Three previous population‐based studies also found that *H. pylori* was associated with an increased risk of dementia,^17^, ^18^, ^19^ whereas one found no association.^20^ Although statistically non‐significant, the latter study reported that the effect estimates between *H. pylori* and dementia increased with age,^20^ while we found strong associations in those aged ≥65 years but a null association in those aged 50 to 64 years. This could indicate an age‐specific effect, which is consistent with other factors that are often conceptualized as affecting dementia risk based on “mid‐life” or “late‐life” exposure.^29^ Alternatively, this may suggest a cohort‐specific effect, as *H. pylori* seroprevalence and associated complications have decreased over time and eradication protocols have only become available in the last few decades.^36^, ^37^ Studies with even longer follow‐up of middle‐aged participants is necessary to explore whether the differential association by age remains, particularly as the number of incident cases in this subgroup in the current study was low (*n* = 265) compared to those in the older age group (*n* = 862). Aside from age, lower socioeconomic status is strongly associated with a greater likelihood of *H. pylori* infection risk.^38^ We found that adjustment for current socioeconomic factors, including educational attainment, occupation and area‐level indicators of material deprivation, did not attenuate the association between *H. pylori* and dementia. We also observed a slightly larger association between *H. pylori* and dementia risk among participants with APOE ε4, the strongest genetic risk factor for Alzheimer's disease. However, the association between *H. pylori* and dementia risk remained similar in APOE ε4 non‐carriers, and there was no interaction between APOE ε4 carrier status, *H. pylori*, and incident dementia risk.

With regard to specific antibodies, CagA and GroEL were associated with an increased risk of incident dementia. CagA is involved in the pathogenesis and virulence of *H. pylori* and is a gastric cancer risk factor, with non‐CagA *H. pylori* strains associated with a weaker cancer risk.^21^ CagA produces a strong inflammatory response and has been implicated in the development of atherosclerosis and large vessel stroke.^39^, ^40^ GroEL is a chaperonin protein that promotes protein folding under environmental stress and has also been linked with gastric cancer risk.^41^, ^42^ It has been hypothesized that GroEL antibodies persist a long time after initial disease‐related infection and represents a particularly suitable marker of past *H. pylori* infection.^42^

Our findings for HSV‐1 and VZV are consistent with a 2019 systematic review and meta‐analysis of 57 studies that also found no association between seropositivity for any herpesviruses and risk of dementia.^43^ More recently, in 1915 participants from the Rotterdam Study, HSV‐1 seropositivity was not associated with dementia risk over an average follow‐up of 9.1 years.^13^ In contrast, in 1002 participants from the Prospective Investigation of Vasculature in Uppsala Seniors cohort, HSV‐1 seropositivity was associated with double the risk of dementia over an average follow‐up of 15 years.^12^ It has been hypothesized that HSV‐1 interacts with APOE ε4, which has been implicated in HSV‐1 infection susceptibility, to increase dementia risk.^44^ Previous longitudinal studies produced mixed evidence and were limited by small sample sizes to perform subgroup analyses.^9^, ^12^, ^14^, ^45^ In the current study, there was a sufficient number of dementia cases in all infectious disease and genetic subgroups for reasonable statistical power, and no evidence of an interaction was found.

The lack of association for HSV‐1 and VZV in the current study might be due to the inability to distinguish between latent and reactivated infection. Analyses of medical records have found that reactivated HSV‐1 and VZV complications, such as herpes zoster and sores, are associated with an increased risk of dementia, with a reduced dementia risk observed in patients administered anti‐viral treatments compared to untreated patients.^46^, ^47^, ^48^ However, selection bias and uncontrolled confounding could explain these associations. Two recent studies minimized these limitations by taking advantage of real‐world changes to shingles vaccination programs in the United States^49^ and Wales,^50^ whereby introduction/discontinuation of vaccinations at specific time points results in a so‐called natural experiment. Both studies found that vaccinations administered to prevent VZV complications were associated with a decreased risk of dementia.^49^, ^50^ Although we found that HSV‐1 reactivation based on self‐reported cold sores was not associated with dementia risk, we observed a non‐significant increased risk of dementia among participants with evidence of VZV reactivation based on self‐reported shingles (HR = 1.74, 95% 0.88 to 3.44). However, these findings should be interpreted with caution as the number of participants with shingles was low (*N* = 44). This is likely due to the question asking participants to report infections they get “once or twice a year,” whereas most affected individuals experience shingles less frequently. We were also unable to account for shingles vaccination, which only a subset of participants near the end of the follow‐up period reported, and there was no information available on anti‐viral medications administration for herpes zoster.

Study strengths include the use of a large and detailed population‐based cohort, with infectious disease serology measures and a long follow‐up period enabling the ascertainment of more than a thousand dementia cases. However, our study also has limitations. Dementia cases were primarily identified in hospital and mortality records which have high accuracy but underestimate cases.^28^ We did not explore dementia subtypes, such as Alzheimer's disease and vascular dementia, as the majority of cases in the cohort were diagnosed after 80 years of age, where mixed pathologies are a common cause of dementia. Changes in dementia diagnosis rates over time might have introduced cohort effects, although the associations remained similar when stratifying by different follow‐up lengths. We were unable to account for several immunosuppressive conditions, such as HIV; however, these are likely to be of low prevalence in this volunteer‐based sample who attended an in‐person assessment. EPIC‐Norfolk participants were exclusively recruited from the East of England in the 1990s, and 99% of the sample in the current study are of white ethnic background. Although we adjusted for a range of factors, residual confounding could remain, particularly factors in childhood, which is when *H. pylori* infection is typically acquired, such as living conditions and socioeconomic status. Finally, reactivation might be the primary driver of herpesvirus associations with dementia, and while we used antibody titer levels and self‐reported information on cold sores and shingles as proxy measures, these likely result in misclassification bias.

In conclusion, we found *H. pylori* seropositivity was consistently associated with an increased risk of dementia even after adjustment for a range of sociodemographic, lifestyle and health‐related factors, and restriction to dementia cases occurring over 15 years later. However, we found no evidence based on serological assessment of antibody levels that past exposure to HSV‐1 and VZV was associated with dementia risk. *H. pylori* might represent a novel target for dementia risk reduction, and future studies investigating the potential mechanistic pathways underlying these associations, and whether they are mediated by other socioeconomic and lifestyle factors, are warranted.

## CONFLICT OF INTEREST STATEMENT

The authors declare no conflicts of interest. Author disclosures are available in the Supporting Information.

## CONSENT STATEMENT

Informed consent was obtained from participants upon recruitment into the EPIC‐Norfolk study.

## Supporting information



ICMJE Disclosures Form

**Supporting Information**: dad270414‐sup‐0002‐SuppMat.docx
