# Supplementary material for: Helicobacter pylori, herpes simplex virus‐1, varicella‐zoster virus, and dementia risk
Source: Alzheimers Dement (Amst). 2026 Jul 6;18(3):e70414. doi: 10.1002/dad2.70414 (PMC13336066; doi:10.1002/dad2.70414)
Supplement: Supplementary file 2 — Supporting Information: dad270414‐sup‐0002‐SuppMat.docx [file DAD2-18-e70414-s001.docx]

**Supplementary Figure 1. Flow chart for participant inclusion**

**
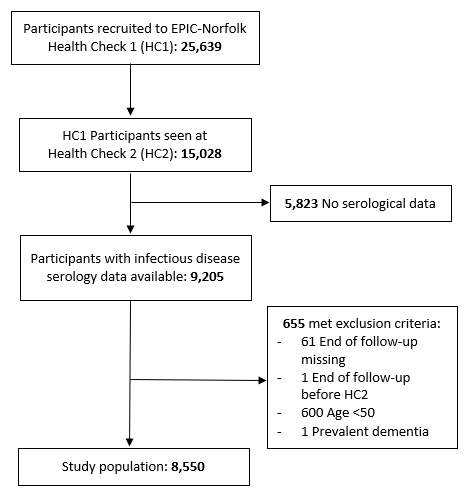
**

The number of participants who meet individual exclusion criteria exceeds the total number excluded (N=655). This is because some participants meet more than one exclusion criteria.

**Supplementary Figure 2. Distribution of age at dementia diagnosis in years**


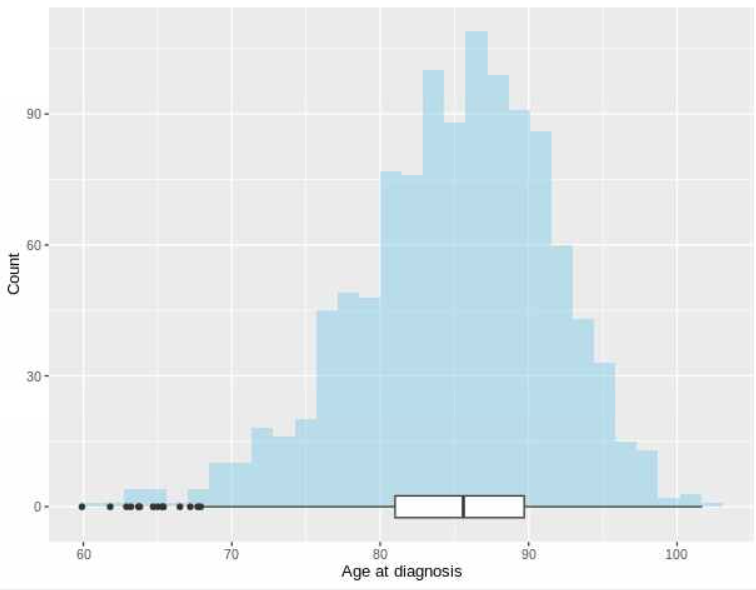


**Supplementary Figure 3. Distribution of time to dementia diagnosis from baseline assesment in years**

**
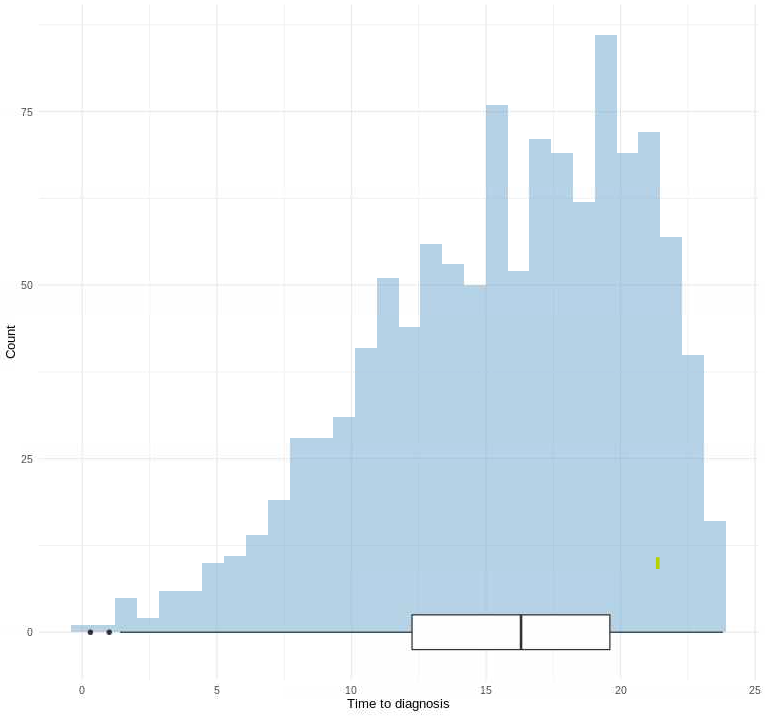
**

**Supplementary Figure 4. *H. pylori,* HSV-1, and VZV antibody level distributions**


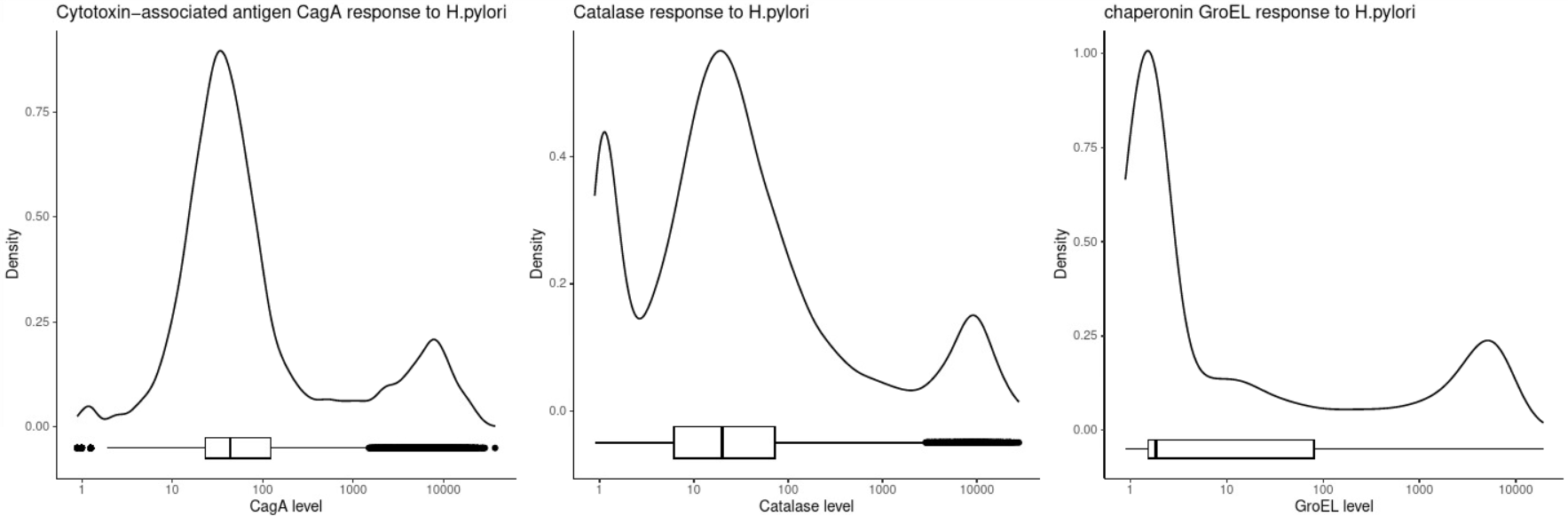

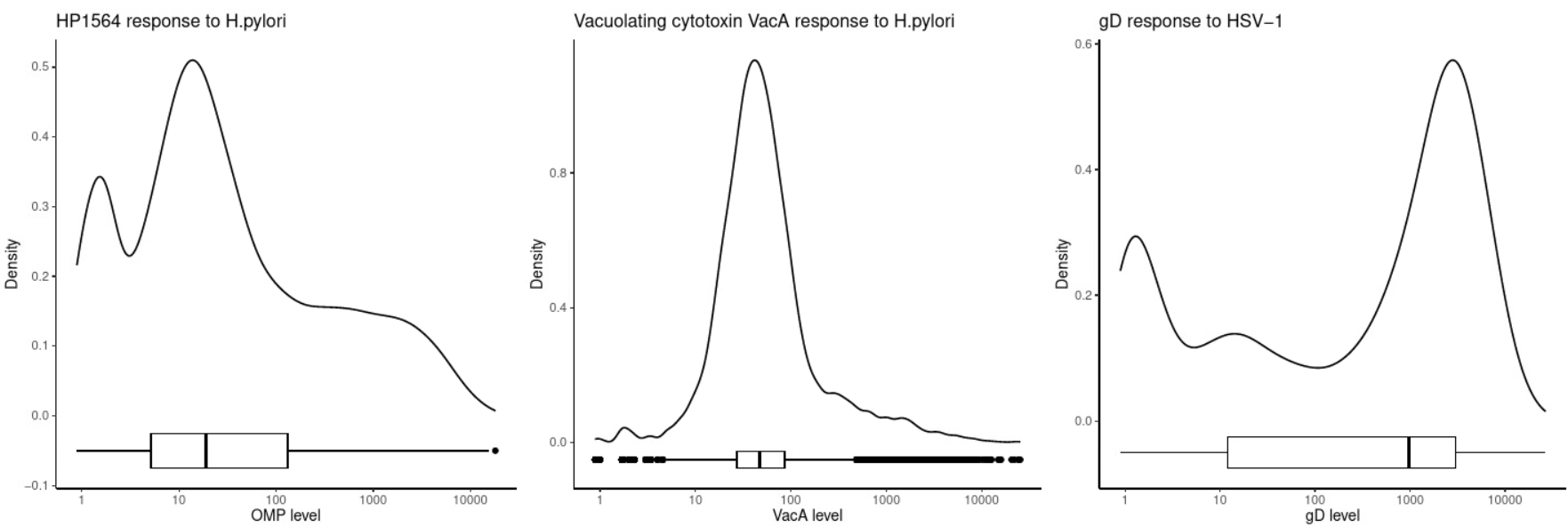

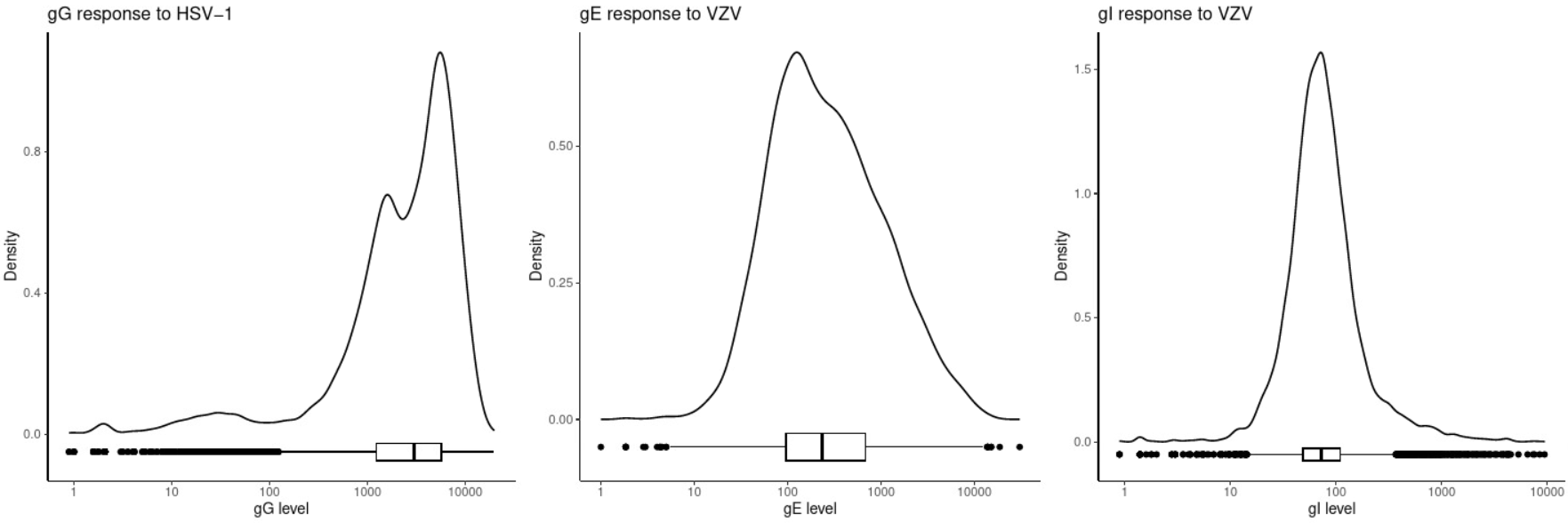


The distribution of each antibody level was based on all seropositive and seronegative participants. The antibody level in log10 scale on the x-axis was used since the distribution of antibody level was widely spread and most observations have small values.

**Supplementary Figure 5. Association between reactivated HSV-1 and VZV and risk of incident dementia in EPIC-Norfolk**


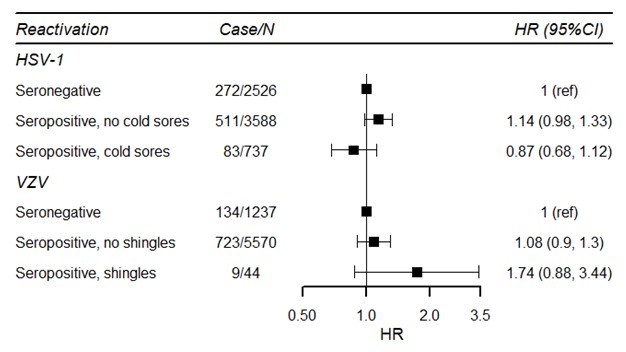


Abbreviations: HR, Hazard Ratio; HSV-1, Herpes Simplex Virus-1; N, Number of Participants; Ref, Reference group; VZV, Varicella-Zoster Virus

Models adjusted for age, sex, education, occupational social class, Townsend deprivation score, body mass index, smoking status, alcohol drinking status, physical activity, hypertension and diabetes

**Supplementary Table 1. Overview of infectious disease antibodies and definition of seropositivity**

| **Infectious disease** | | **Antibodies** | **Antibody MFI cut-point** | **Definition of Seropositivity based on MFI cut-points** |
| --- | --- | --- | --- | --- |
| ***Primary exposures*** | | | | |
| Herpes simplex virus-1 (HSV-1) | anti-gD response to Herpes simplex virus 1 | | 100 | 2 out of 2 antibodies above cut-points |
|  | anti-gG response to Herpes simplex virus 1 | | 100 |  |
| Varicella zoster virus (VZV) | gE response to Varicella zoster virus | | 80 | 1 out of 2 antibodies above cut-points |
|  | gI response to Varicella zoster virus | | 100 |  |
| *Helicobacter pylori* (*H. pylori*) | Cytotoxin-associated antigen CagA response to Helicobacter pylori | | 400 | 2 out of 5 antibodies above cut-points |
|  | Catalase response to Helicobacter pylori | | 180 |  |
|  | Chaperonin GroEL response to Helicobacter pylori | | 60 |  |
|  | HP1564 response to Helicobacter pylori | | 170 |  |
|  | Vacuolating cytotoxin VacA response to Helicobacter pylori | | 80 |  |
| ***Secondary exposures*** | | | | |
| Herpes simplex virus-2 (HSV-2) | anti-gD (85%aaidentity) response to Herpes simplex virus 2 | | 100 | 2 out of 2 antibodies above cut-points |
|  | anti-gG (<30%aaidentity) response to Herpes simplex virus 2 | | 100 |  |
| Epstein-Barr virus (EBV) | Early Antigen-Diffuse response to Epstein-Barr virus | | 100 | 2 out of 4 antibodies above cut-points |
|  | Epstein Barr nucleic antigen 1 response to Epstein-Barr virus | | 250 |  |
|  | Viral Capsid Antigen response to Epstein-Barr virus | | 250 |  |
|  | Z Epstein Barr replication activator response to Epstein-Barr virus | | 100 |  |
| Human cytomegalovirus (HCMV) | Tegument Protein p150 response to Human Cytomegalovirus | | 150 | 2 out of 4 antibodies above cut-points |
|  | Tegument Protein p28 response to Human Cytomegalovirus | | 150 |  |
|  | Gene product p52 (UL44) response to Human Cytomegalovirus | | 150 |  |
|  | Tegument Protein p65 response to Human Cytomegalovirus | | 150 |  |
| Human herpes virus 6a (HHV6a) | Immediate early protein HHV6A response to Human Herpes virus 6 | | 50 | 1 out of 1 antibody above cut-point |
| Human herpes virus 6b (HHV6b) | Immediate early protein HHV6B response to Human Herpes virus 6 | | 50 | 1 out of 1 antibody above cut-point |
| Human herpes virus 6 (HHV6) | Major Capsid Protein response to Human Herpesvirus 6 | | 100 | 2 out of 4 antibodies above cut-points |
|  | Protein p100 response to Human Herpesvirus 6 | | 50 |  |
|  | Protein p101 K response to Human Herpesvirus 6 | | 50 |  |
|  | Gene U94 response to Human Herpes virus 6 | | 100 |  |
| Human herpes virus 7 (HHV7) | Glycoprotein B response to Human Herpes virus 7 | | 50 | 2 out of 2 antibodies above cut-points |
|  | Gene U14 response to Human Herpes virus 7 | | 100 |  |
| *Toxoplasma gondii* (*T. gondii*) | Protein p22 response to Toxoplasma gondii | | 50 | 2 out of 2 antibodies above cut-points |
|  | Tachyzoite surface antigen-1 response to Toxoplasma gondii | | 50 |  |

Abbreviations: MFI, median fluorescence intensities

**Supplementary Table 2. ICD-10 codes used to determine all-cause dementia**

| **ICD-10 code** | **Diagnosis** |
| --- | --- |
| F00 | Dementia in Alzheimer's disease |
| F00.0 | Dementia in Alzheimer's disease with early onset |
| F00.1 | Dementia in Alzheimer's disease with late onset |
| F00.2 | Dementia in Alzheimer's disease, atypical or mixed type |
| F00.9 | Dementia in Alzheimer's disease, unspecified |
| F01 | Vascular dementia |
| F01.0 | Vascular dementia of acute onset |
| F01.1 | Multi-infarct dementia |
| F01.2 | Subcortical vascular dementia |
| F01.8 | Other vascular dementia |
| F01.9 | Vascular dementia, unspecified |
| F02 | Dementia in other diseases classified elsewhere |
| F02.0 | Dementia in Picks disease |
| F02.1 | Dementia in Creutzfeldt-Jacob disease |
| F02.2 | Dementia in Huntington’s disease |
| F02.3 | Dementia in Parkinson’s disease |
| F02.8 | Dementia in other specified diseases classified elsewhere |
| F03 | Unspecified dementia |
| F05.1 | Delirium superimposed on dementia |
| G30 | Alzheimer’s disease |
| G30.0 | Alzheimer’s disease with early onset |
| G30.1 | Alzheimer’s disease with late onset |
| G30.8 | Other Alzheimer's disease |
| G30.9 | Alzheimer's disease unspecified |
| G31.0 | Circumscribed brain atrophy |
| G31.8 | Other specified degenerative diseases of nervous system |

Abbreviations: ICD, International Classification of Diseases

**Supplementary Table 3. Association between *H. pylori, HSV-1, and, VZV* serostatus and risk of incident all-cause dementia with sequential adjustment for covariates in EPIC-Norfolk**

|  | **Cases/N** | **HR (95% CI)** |
| --- | --- | --- |
| ***H.pylori*** |  |  |
| Seronegative | 726/6262 | 1 (ref) |
| Seropositive (Unadjusted) | 401/2288 | 1.83 (1.62, 2.07) |
| +age |  | 1.26 (1.12, 1.43) |
| +sex |  | 1.25 (1.11, 1.42) |
| +education |  | 1.24 (1.09, 1.40) |
| +occupational social class |  | 1.23 (1.09, 1.39) |
| +Townsend deprivation score |  | 1.23 (1.09, 1.40) |
| +body mass index |  | 1.24 (1.09, 1.40) |
| +smoking status |  | 1.23 (1.08, 1.39) |
| +alcohol drinking status |  | 1.25 (1.10, 1.41) |
| +physical activity |  | 1.24 (1.09, 1.41) |
| +hypertension |  | 1.24 (1.09, 1.41) |
| +diabetes |  | 1.24 (1.09, 1.41) |
| **HSV-1** |  |  |
| Seronegative | 358/3070 | 1 (ref) |
| Seropositive (Unadjusted) | 769/5480 | 1.30 (1.14, 1.47) |
| +age |  | 1.05 (0.93, 1.19) |
| +sex |  | 1.05 (0.93, 1.19) |
| +education |  | 1.04 (0.91, 1.18) |
| +occupational social class |  | 1.03 (0.91, 1.17) |
| +Townsend deprivation score |  | 1.03 (0.91, 1.17) |
| +body mass index |  | 1.04 (0.91, 1.18) |
| +smoking status |  | 1.03 (0.91, 1.17) |
| +alcohol drinking status |  | 1.04 (0.91, 1.18) |
| +physical activity |  | 1.04 (0.91, 1.18) |
| +hypertension |  | 1.04 (0.92, 1.19) |
| +diabetes |  | 1.03 (0.91, 1.18) |
| **VZV** |  |  |
| Seronegative | 179/1495 | 1 (ref) |
| Seropositive (Unadjusted) | 948/7055 | 1.15 (0.98, 1.35) |
| +age |  | 1.07 (0.91, 1.26) |
| +sex |  | 1.06 (0.90, 1.25) |
| +education |  | 1.06 (0.90, 1.24) |
| +occupational social class |  | 1.06 (0.90, 1.24) |
| +Townsend deprivation score |  | 1.06 (0.90, 1.24) |
| +body mass index |  | 1.06 (0.91, 1.25) |
| +smoking status |  | 1.06 (0.91, 1.25) |
| +alcohol drinking status |  | 1.03 (0.87, 1.21) |
| +physical activity |  | 1.02 (0.87, 1.20) |
| +hypertension |  | 1.02 (0.87, 1.20) |
| +diabetes |  | 1.01 (0.86, 1.19) |

Abbreviations: CI, Confidence Interval; HR, Hazard Ratio; HSV-1, Herpes Simplex Virus-1; *H. Pylori*, *Helicobacter Pylori*; Ref, Reference group; VZV, Varicella-Zoster Virus

**Supplementary Table 4 – Association between covariates included in the fully adjusted model and risk of incident all-cause dementia in EPIC-Norfolk**

| **Characteristics** | **HR (95% CI)** |
| --- | --- |
| Age | 1.17 (1.16, 1.18) |
| Men | 1.05 (0.92, 1.21) |
| Educational attainment |  |
| Degree or equivalent | 1 (Reference) |
| Upper secondary school (A-Level) or equivalent | 1.30 (1.04, 1.64) |
| Lower secondary school (O-Level) or equivalent | 1.24 (0.93, 1.66) |
| Less than lower secondary school (O-level) or  no qualifications | 1.23 (0.97, 1.56) |
| Townsend deprivation score, quintile |  |
| 1 (least deprived) | 1 (Reference) |
| 2 | 1.20 (0.99, 1.45) |
| 3 | 0.97 (0.80, 1.19) |
| 4 | 1.16 (0.96, 1.41) |
| 5 (most deprived) | 1.04 (0.85, 1.26) |
| Occupational social class |  |
| l – Professionals | 1 (Reference) |
| ll – Managerial and technical | 1.00 (0.76, 1.30) |
| lllN + lllM – Non-manual and  manual skilled | 1.00 (0.76, 1.31) |
| IV – Partly skilled | 1.07 (0.79, 1.46) |
| V – Unskilled | 1.31 (0.87, 1.96) |
| BMI |  |
| <25 | 1 (Reference) |
| 25-29.9 | 1.02 (0.40, 2.62) |
| ≥30 | 1.14 (0.56, 2.31) |
| Smoking status |  |
| Never | 1 (Reference) |
| Former | 1.15 (1.00, 1.31) |
| Current | 1.29 (0.97, 1.72) |
| Alcohol drinking status |  |
| Never | 1 (Reference) |
| Former | 0.96 (0.73, 1.27) |
| Current | 0.90 (0.70, 1.15) |
| Physical activity |  |
| Active | 1 (Reference) |
| Moderately active | 0.91 (0.76, 1.08) |
| Moderately inactive | 0.91 (0.77, 1.06) |
| Inactive | 1.24 (1.00, 1.53) |
| Hypertension | 1.14 (1.00, 1.31) |
| Diabetes | 1.86 (1.40, 2.48) |

Abbreviations: BMI, Body Mass Index, CI, Confidence Interval; HR, Hazard Ratio; HSV-1, Herpes Simplex Virus-1

**Supplementary Table 5 - Association between H. pylori, HSV-1, and, VZV serostatus and risk of incident all-cause dementia by birth year in EPIC-Norfolk**

| **Birth Year** | **Serostatus** | **Cases/N** | **HR (95% CI)** |
| --- | --- | --- | --- |
| ***H. Pylori*** |  |  |  |
| <1930 | Seronegative | 354/1493 | 1 (ref) |
|  | Seropositive | 240/914 | 1.22 (1.03, 1.44) |
| 1930-1939 | Seronegative | 299/2237 | 1 (ref) |
|  | Seropositive | 148/857 | 1.33 (1.09, 1.63) |
| ≥1940 | Seronegative | 73/2532 | 1 (ref) |
|  | Seropositive | 13/517 | 0.77 (0.40, 1.46) |
| **HSV-1** |  |  |  |
| <1930 | Seronegative | 164/730 | 1 (ref) |
|  | Seropositive | 430/1677 | 1.15 (0.95, 1.39) |
| 1930-1939 | Seronegative | 161/1089 | 1 (ref) |
|  | Seropositive | 286/2005 | 0.96 (0.78, 1.17) |
| ≥1940 | Seronegative | 33/1251 | 1 (ref) |
|  | Seropositive | 53/1798 | 1.02 (0.65, 1.59) |
| **VZV** |  |  |  |
| <1930 | Seronegative | 97/395 | 1 (ref) |
|  | Seropositive | 497/2012 | 0.98 (0.78, 1.23) |
| 1930-1939 | Seronegative | 70/518 | 1 (ref) |
|  | Seropositive | 377/2576 | 1.01 (0.78, 1.31) |
| ≥1940 | Seronegative | 12/582 | 1 (ref) |
|  | Seropositive | 74/2467 | 1.63 (0.86, 3.10) |

Abbreviations: CI, Confidence Interval; HR, Hazard Ratio; HSV-1, Herpes Simplex Virus-1; H. Pylori, Helicobacter Pylori; N, Number of Participants; Ref, Reference group; VZV, Varicella-Zoster Virus

Models adjusted for age, sex, education, occupational social class, Townsend deprivation score, body mass index, smoking status, alcohol drinking status, physical activity, hypertension and diabetes

p-value for interaction between birth year and *H. pylori* = 0.166, HSV-1 = 0.547, VZV = 0.326

**Supplementary Table 6 - Association between H. pylori, HSV-1 , and VZV serostatus and risk of incident all-cause dementia by sex in EPIC-Norfolk**

| **Sex** | **Serostatus** | **Cases/N** | **HR (95% CI)** |
| --- | --- | --- | --- |
| ***H. Pylori*** |  |  |  |
| Female | Seronegative | 464/3754 | 1 (ref) |
|  | Seropositive | 212/1167 | 1.19 (1.00, 1.40) |
| Male | Seronegative | 262/2508 | 1 (ref) |
|  | Seropositive | 189/1121 | 1.35 (1.11, 1.64) |
| **HSV-1** |  |  |  |
| Female | Seronegative | 213/1726 | 1 (ref) |
|  | Seropositive | 463/3195 | 1.03 (0.87, 1.22) |
| Male | Seronegative | 145/1344 | 1 (ref) |
|  | Seropositive | 306/2285 | 1.05 (0.85, 1.29) |
| **VZV** |  |  |  |
| Female | Seronegative | 115/987 | 1 (ref) |
|  | Seropositive | 561/3934 | 1.10 (0.89, 1.34) |
| Male | Seronegative | 64/508 | 1 (ref) |
|  | Seropositive | 387/3121 | 0.87 (0.66, 1.14) |

Abbreviations: CI, Confidence Interval; HR, Hazard Ratio; HSV-1, Herpes Simplex Virus-1; H. Pylori, Helicobacter Pylori; N, Number of Participants; Ref, Reference group; VZV, Varicella-Zoster Virus

Models adjusted for age, sex, education, occupational social class, Townsend deprivation score, body mass index, smoking status, alcohol drinking status, physical activity, hypertension and diabetes

p-value for interaction between sex and *H. pylori* = 0.350, HSV-1 = 0.770, VZV = 0.213

**Supplementary Table 7. Association between other infectious diseases and risk of incident all-cause dementia in EPIC-Norfolk**

|  | **Cases** | **HR (95% CI)** | **p-value for linear trend** |
| --- | --- | --- | --- |
| **Herpes simplex virus-2** | |  | |
| Seronegative | 1098/8255 | 1 (ref) |  |
| Seropositive | 29/295 | 0.77 (0.53, 1.12) |  |
| **Anti-gD response** | |  | 0.62 |
| Seronegative | 518/4052 | 1 (ref) |  |
| Seropositive T1 | 216/1500 | 1.00 (0.85, 1.18) |  |
| Seropositive T2 | 194/1499 | 0.92 (0.78, 1.09) |  |
| Seropositive T3 | 199/1499 | 0.99 (0.84, 1.17) |  |
| **Anti-gG response** | |  | 0.21 |
| Seronegative | 1094/8223 | 1 (ref) |  |
| Seropositive T1 | 12/109 | 0.87 (0.49, 1.53) |  |
| Seropositive T2 | 11/109 | 0.69 (0.38, 1.25) |  |
| Seropositive T3 | 10/109 | 0.84 (0.45, 1.57) |  |
| **Epstein-Barr virus** | |  | |
| Seronegative | 103/740 | 1 (ref) |  |
| Seropositive | 1024/7810 | 0.87 (0.71, 1.08) |  |
| **Early Antigen-Diffuse response** | |  | 0.09 |
| Seronegative | 211/1516 | 1 (ref) |  |
| Seropositive T1 | 313/2345 | 0.92 (0.77, 1.09) |  |
| Seropositive T2 | 328/2345 | 0.95 (0.80, 1.13) |  |
| Seropositive T3 | 275/2344 | 0.84 (0.70, 1.00) |  |
| **Epstein-Barr nucleic antigen1 response** | |  | 0.63 |
| Seronegative | 95/710 | 1 (ref) |  |
| Seropositive T1 | 359/2614 | 0.91 (0.72, 1.14) |  |
| Seropositive T2 | 326/2613 | 0.87 (0.69, 1.10) |  |
| Seropositive T3 | 347/2613 | 0.91 (0.72, 1.15) |  |
| **Viral Capsid Antigen response** | |  | 0.76 |
| Seronegative | 101/742 | 1 (ref) |  |
| Seropositive T1 | 319/2603 | 0.84 (0.67, 1.06) |  |
| Seropositive T2 | 336/2603 | 0.86 (0.68, 1.08) |  |
| Seropositive T3 | 371/2602 | 0.93 (0.74, 1.16) |  |
| **Z Epstein-Barr replication activator response** | | | 0.97 |
| Seronegative | 174/1317 | 1 (ref) |  |
| Seropositive T1 | 308/2411 | 0.90 (0.75, 1.10) |  |
| Seropositive T2 | 314/2411 | 1.00 (0.82, 1.21) |  |
| Seropositive T3 | 331/2411 | 0.95 (0.79, 1.15) |  |
| **Human cytomegalovirus** | |  |  |
| Seronegative | 404/3562 | 1 (ref) |  |
| Seropositive | 723/4988 | 1.08 (0.95, 1.23) |  |
| **Tegument Protein p150 response** | |  | 0.20 |
| Seronegative | 445/3858 | 1 (ref) |  |
| Seropositive T1 | 209/1564 | 1.05 (0.89, 1.24) |  |
| Seropositive T2 | 233/1564 | 1.08 (0.92, 1.28) |  |
| Seropositive T3 | 240/1564 | 1.10 (0.93, 1.29) |  |
| **Tegument Protein p28 response** | |  | 0.37 |
| Seronegative | 434/3765 | 1 (ref) |  |
| Seropositive T1 | 240/1595 | 1.06 (0.90, 1.24) |  |
| Seropositive T2 | 207/1595 | 0.95 (0.80, 1.12) |  |
| Seropositive T3 | 246/1595 | 1.12 (0.96, 1.32) |  |
| **Gene product p52 (UL44) response** | |  | 0.02 |
| Seronegative | 359/3233 | 1 (ref) |  |
| Seropositive T1 | 208/1773 | 0.94 (0.79, 1.12) |  |
| Seropositive T2 | 272/1772 | 1.19 (1.01, 1.40) |  |
| Seropositive T3 | 288/1772 | 1.15 (0.98, 1.35) |  |
| **Tegument Protein p65 response** | |  | 0.63 |
| Seronegative | 503/4250 | 1 (ref) |  |
| Seropositive T1 | 203/1434 | 1.03 (0.87, 1.22) |  |
| Seropositive T2 | 209/1433 | 1.03 (0.88, 1.22) |  |
| Seropositive T3 | 212/1433 | 1.04 (0.88, 1.22) |  |
| **Human herpes virus 6a** | |  |  |
| Seronegative | 1001/7664 | 1 (ref) |  |
| Seropositive | 126/886 | 1.10 (0.91, 1.32) |  |
| **immediate early protein HHV6A response** | |  | 0.24 |
| Seronegative | 382/2881 | 1 (ref) |  |
| Seropositive T1 | 256/1890 | 0.95 (0.81, 1.12) |  |
| Seropositive T2 | 253/1890 | 0.94 (0.80, 1.11) |  |
| Seropositive T3 | 236/1889 | 0.91 (0.77, 1.07) |  |
| **Human herpes virus 6b** | |  |  |
| Seronegative | 902/6692 | 1 (ref) |  |
| Seropositive | 225/1858 | 0.93 (0.80, 1.08) |  |
| **immediate early protein HHV6B response** | |  | 0.23 |
| Seronegative | 285/2036 | 1 (ref) |  |
| Seropositive T1 | 289/2172 | 0.94 (0.80, 1.11) |  |
| Seropositive T2 | 285/2171 | 0.98 (0.83, 1.16) |  |
| Seropositive T3 | 268/2171 | 0.88 (0.75, 1.05) |  |
| **Human herpes virus 6** | |  |  |
| Seronegative | 409/3005 | 1 (ref) |  |
| Seropositive | 718/5545 | 0.93 (0.82, 1.05) |  |
| **Major Capsid Protein response** | |  | 0.79 |
| Seronegative | 1035/7889 | 1 (ref) |  |
| Seropositive T1 | 26/221 | 0.85 (0.57, 1.25) |  |
| Seropositive T2 | 33/220 | 1.01 (0.71, 1.43) |  |
| Seropositive T3 | 33/220 | 0.98 (0.69, 1.39) |  |
| **protein p100 response** | |  | 0.11 |
| Seronegative | 977/7493 | 1 (ref) |  |
| Seropositive T1 | 46/353 | 0.95 (0.71, 1.28) |  |
| Seropositive T2 | 53/352 | 1.18 (0.89, 1.56) |  |
| Seropositive T3 | 51/352 | 1.23 (0.92, 1.64) |  |
| **protein p101K response** | |  | 0.16 |
| Seronegative | 852/6267 | 1 (ref) |  |
| Seropositive T1 | 96/761 | 1.04 (0.84, 1.28) |  |
| Seropositive T2 | 88/761 | 0.93 (0.75, 1.17) |  |
| Seropositive T3 | 91/761 | 0.85 (0.68, 1.06) |  |
| **geneU94 response** | |  | 0.28 |
| Seronegative | 1037/7792 | 1 (ref) |  |
| Seropositive T1 | 25/253 | 0.88 (0.59, 1.31) |  |
| Seropositive T2 | 35/253 | 1.16 (0.83, 1.63) |  |
| Seropositive T3 | 30/252 | 0.74 (0.51, 1.07) |  |
| **Human herpes virus 7** | |  |  |
| Seronegative | 242/1612 | 1 (ref) |  |
| Seropositive | 885/6938 | 0.86 (0.74, 0.99) |  |
| **Glyco protein B response** | |  | 0.13 |
| Seronegative | 113/853 | 1 (ref) |  |
| Seropositive T1 | 346/2566 | 1.00 (0.80, 1.24) |  |
| Seropositive T2 | 344/2566 | 0.98 (0.79, 1.22) |  |
| Seropositive T3 | 324/2565 | 0.88 (0.71, 1.10) |  |
| **Gene U14 response** | |  | 0.12 |
| Seronegative | 168/957 | 1 (ref) |  |
| Seropositive T1 | 329/2531 | 0.85 (0.70, 1.02) |  |
| Seropositive T2 | 308/2531 | 0.81 (0.67, 0.98) |  |
| Seropositive T3 | 322/2531 | 0.84 (0.69, 1.01) |  |
| **Toxoplasma gondii** | |  | |
| Seronegative | 997/7616 | 1 (ref) |  |
| Seropositive | 130/934 | 0.90 (0.75, 1.09) |  |
| **proteinp22 response** | |  | 0.78 |
| Seronegative | 950/7226 | 1 (ref) |  |
| Seropositive T1 | 51/442 | 0.73 (0.55, 0.98) |  |
| Seropositive T2 | 59/441 | 0.89 (0.68, 1.17) |  |
| Seropositive T3 | 67/441 | 1.10 (0.85, 1.43) |  |
| **Tachyzoite surface antigen-1 response** | |  | 0.17 |
| Seronegative | 798/6202 | 1 (ref) |  |
| Seropositive T1 | 106/783 | 0.93 (0.75, 1.14) |  |
| Seropositive T2 | 112/783 | 0.98 (0.80, 1.19) |  |
| Seropositive T3 | 111/782 | 0.86 (0.70, 1.05) |  |

Abbreviations: CI, Confidence Interval; HR, Hazard Ratio; Ref, Reference group; T, Tertile

Models adjusted for age, sex, education, occupation social class, socioeconomic status, body mass index, smoking status, alcohol drinker status, physical activity, hypertension and diabetes
